# Hampi Tariki kit: thin-layer chromatography for semiquantitative screening of solid oral medicines in Peru

**DOI:** 10.17843/rpmesp.2026.433.15446

**Published:** 2026-08-28

**Authors:** Gladys M. Mónico-Rosales, Fredy R. Mostacero-Rodríguez, B. Rosa Quispe-Paitán, E. Javier Rodríguez-Calzado, Juan J. Ortiz-Bernaola, Alejandro Nuñez-Galindo

**Affiliations:** 1 Centro Nacional de Control de Calidad, Instituto Nacional de Salud, Lima, Perú Centro Nacional de Control de Calidad Instituto Nacional de Salud Lima Perú

**Keywords:** Counterfeit Drugs, Thin-Layer Chromatography, Quality Control, Health Surveillance, Pharmaceutical Technology

## Abstract

**Objectives.:**

To develop and validate rapid thin-layer chromatography (TLC) methods integrated into the Hampi Tariki kit for the semiquantitative screening of active pharmaceutical ingredients (APIs) in oral solid medications marketed in Peru.

**Materials and methods.:**

Eight TLC methods were developed and validated for tablets and capsules containing acyclovir, dicloxacillin, dimenhydrinate, ibuprofen, diclofenac sodium, paracetamol, norfloxacin, phenazopyridine hydrochloride, chlorphenamine maleate, and phenylephrine hydrochloride. Fifty-seven commercial samples available in the national market, from different manufacturing laboratories, including branded and generic products previously verified as compliant by official methods at the Centro Nacional de Control de Calidad (CNCC) of the Instituto Nacional de Salud (INS), were evaluated. Silica gel 60 F254 plates, reference standards, and analytical grade solvents were used. Validation was performed according to ICH Q2(R1) guideline, evaluating selectivity, linearity, precision, accuracy, visual detection limit, and robustness.

**Results.:**

The methods allowed for visual and densitometric differentiation of levels equivalent to 80%, 100%, and 120% of the declared content. Retention factor (Rf) values were between 0.20 and 0.85, and multicomponent formulations presented chromatographic resolution ≥1.0. Correlation coefficients were greater than 0.99 for system and method linearity; repeatability showed coefficients of variation less than 3.0%, intermediate precision less than 5.0%, and bias against high-performance liquid chromatography (HPLC) remained within the ±3.00% range. Deliberate variations evaluated in robustness did not produce significant changes in Rf values.

**Conclusions.:**

The developed methods demonstrated adequate analytical performance as rapid semiquantitative screening tests. Integrated into the Hampi Tariki kit, they constitute a proposal with potential to support the preliminary identification of suspicious samples and prioritize their confirmation by HPLC or other official methods.

## INTRODUCTION

Counterfeit medicines represent a relevant public health problem because they may contain incorrect ingredients, a total or partial absence of the active pharmaceutical ingredient (API), concentrations different from those declared, counterfeit packaging, or fraudulent information about their identity, composition, or origin ^1^. These products can cause therapeutic failures, adverse events, loss of trust in healthcare systems, and, in the case of anti-infectives, contribute to antimicrobial resistance ^1^^-^^3^.

In Peru, drug counterfeiting constitutes a documented problem. Medina *et al.* analyzed alerts issued by the General Directorate of Medicines, Supplies and Drugs (DIGEMID) between 1997 and 2014, and identified 1738 cases of counterfeit medicines; 58.1% were associated with pharmaceutical establishments and 20.1% with non-pharmaceutical commercial establishments ^1^. Additionally, tablets were the most frequent pharmaceutical form, and analgesics and systemic antibacterials were among the most affected therapeutic groups ^1^. More recently, Gutiérrez-García *et al*. analyzed national counterfeiting alerts issued between 2013 and 2024, and identified 1247 counterfeit pharmaceutical products, predominantly brand-name medicines, oral solid forms, and frequently used products such as analgesics, antipyretics, anti-inflammatories, vitamins, and antibacterials ^2^.

The health surveillance of these products requires reliable confirmatory methods, such as high-performance liquid chromatography (HPLC, for its acronym in English) or other pharmacopoeial procedures. However, these analyses demand equipped laboratories, specialized personnel, analytical grade reagents, and response times that may limit their immediate application in decentralized interventions ^4^^-^^7^. Therefore, rapid screening technologies have been proposed as complementary tools to guide initial decisions and prioritize suspicious samples, especially in scenarios where immediate access to an instrumental laboratory is limited ^5^^-^^7^.

Thin-layer chromatography (TLC), used in initiatives such as the Global Pharma Health Fund Minilab (GPHF-Minilab), allows for the evaluation of the API identity and a semiquantitative estimation by comparing the size, color, intensity, and retention factor (Rf) of the sample spots against reference materials ^8^^-^^13^. This low-cost technology has shown utility for recognizing products with absence of API, incorrect ingredient, or markedly low content; however, it does not replace official confirmatory methods nor does it allow, by itself, to establish full pharmacopoeial compliance ^9^^-^^15^.

In this context, it is relevant to have rapid TLC methods adapted to oral solid drugs of interest for health surveillance in Peru. Therefore, the objective of the study was to develop and validate rapid TLC analytical methods for the semi-quantitative screening of APIs in oral solid drugs integrated into the Hampi Tariki kit.

**KEY MESSAGES**

Motivation for study. Counterfeit medicines constitute a public health problem in Peru, as they can cause therapeutic failures, adverse reactions, and antimicrobial resistance. Health surveillance requires rapid, portable, and accessible tools.

Main findings. Eight thin-layer chromatography methods, integrated into the Hampi Tariki kit, were developed and validated to detect active pharmaceutical ingredients in oral solid medications. The methods differentiated contents equivalent to 80%, 100%, and 120% of the declared amount. The validated methods demonstrated their analytical performance in accordance with international guidelines.

Public health implications. The kit allows preliminary identification of suspicious samples and prioritization of their confirmatory analysis, as a complement and not a substitute for official pharmacopeial methods.

## MATERIALS AND METHODS

An analytical study was conducted for the development and validation of rapid TLC methods for the semi-quantitative screening of APIs in oral solid dosage forms.

### Pharmaceutical samples

The study population consisted of drugs registered with DIGEMID and marketed in Peru, in solid oral dosage forms: tablets and capsules. Monocomponent formulations of acyclovir, dicloxacillin, dimenhydrinate, ibuprofen, and sodium diclofenac were included, as well as combinations of sodium diclofenac/paracetamol, norfloxacin/phenazopyridine hydrochloride, and chlorphenamine maleate/phenylephrine hydrochloride/paracetamol.

The samples corresponded to commercial brand and generic drugs, from different manufacturing laboratories, concentrations, and commercial formulations available in the national market. In total, 57 commercial matrices were evaluated: acyclovir from four manufacturing laboratories, dicloxacillin from seven, dimenhydrinate from five, ibuprofen from ten, diclofenac sodium from six, diclofenac sodium/paracetamol from ten, norfloxacin/phenazopyridine hydrochloride from eight, and chlorphenamine maleate/phenylephrine hydrochloride/paracetamol from seven.

All samples used for validation were previously evaluated using official quality control methods, including HPLC when applicable, and had compliant results. Therefore, the samples used did not correspond to real counterfeit medicines or seized products, but to compliant products used for the development, optimization, and analytical validation of the methods.

The selection of the APIs was based on national background information describing drug counterfeiting in oral solid forms and in frequently used therapeutic groups, such as analgesics, anti-inflammatories, antibacterials, and symptomatic combinations ^1^^,^^2^. Additionally, the availability of these products in the national market and their relevance for health surveillance were considered.

### Reagents and standards

USP (United States Pharmacopeia*,* United States) reference standards of acyclovir, chlorphenamine maleate, diclofenac sodium, dicloxacillin, dimenhydrinate, phenazopyridine hydrochloride, phenylephrine hydrochloride, ibuprofen, paracetamol, and norfloxacin were used.

Methanol, ethyl acetate, toluene, and 25% ammonia solution were obtained from Merck (Darmstadt, Germany), and glacial acetic acid from J.T. Baker (Avantor, United States). The Milli-Q Advantage A10 purification system (Millipore, France) was used to produce ultrapure water. During development, the use of solvents with lower relative environmental impact was prioritized, avoiding the use of benzene, chloroform, and formic acid. When analytically viable, chloroform was replaced by toluene and formic acid by glacial acetic acid.

### Development and Optimization of the Methods by TLC

The development of the methods was based on the GPHF-Minilab TLC assay model and on previously published methods when available ^8^^,^^13^^,^^16^^-^^21^. For APIs or combinations not covered by the literature, different solvent mixtures were evaluated considering the polarity of the components, the lipophilicity of the APIs, and the need to obtain suitable Rf values for visual and densitometric reading.

During the methodological development, different combinations of solvents for the mobile phase and different sample diluents were tested. The final selection considered adequate Rf values, defined spots, appropriate API recovery, and operational stability of the solutions during the assay run time. Under rapid screening conditions, the complete application of the methodologies takes approximately two hours; within this analytical window, no visible changes were observed that would impede the reading of Rf, intensity comparison, or semi-quantitative interpretation. This observation corresponds to operational stability during method use and does not replace a formal stability study under controlled storage conditions.

A chromatographic system was considered acceptable when it produced Rf values between 0.20 and 0.85, defined spots and, in multicomponent formulations, resolution equal to or greater than 1.0 between APIs or against possible interferents. In multicomponent systems, the evaluation was limited to the commercial combinations included in the study, therefore the results should not be extrapolated to other API combinations without additional experimental verification.

Silica gel 60 F254 chromatographic plates with aluminum support, 5 × 10 cm and 20 × 10 cm, were developed in chromatographic chambers saturated with filter paper. Solutions were applied 1.5 cm from the lower edge of the plate. The mobile phase development distance was 7.5 cm and the selected saturation time was 15 minutes, after evaluating times of 12, 15, and 18 minutes. Visualization was performed under ultraviolet (UV) light at 254 nm or 366 nm, according to the API, and by development with iodine vapors when it corresponded to the method.

In the case of dimenhydrinate, the method was oriented to the chromatographic monitoring of diphenhydramine as the main marker. Although dimenhydrinate corresponds to a salt formed by diphenhydramine and 8-chlorotheophylline, the latter was not validated as an independent marker in the present study. Therefore, the scope of the method should be interpreted as semiquantitative screening of the diphenhydramine component in dimenhydrinate formulations.

### Semi-quantitative interpretation criterion

Semiquantitative evaluation was based on the comparison of the Rf, size, color, and intensity of the sample spot against reference materials prepared at concentrations equivalent to 80%, 100%, and 120% of the declared content of the API. For visual reading, a sample was considered compatible with the expected content when the spot showed the same Rf and an intensity comparable to the 100% reference, without being lower than the 80% reference or showing additional spots attributable to undeclared APIs or relevant interferents.

Samples with spots of intensity less than the 80% reference, absence of the spot corresponding to the API, Rf not concordant with the standard, presence of unexplained additional spots or deficient separation were considered suspicious and would require confirmation by official methods, such as HPLC or other pharmacopeial procedures. This criterion does not constitute a definitive quantification of the API content, but rather a semi-quantitative estimation aimed at preliminary screening ^8^^-^^13^.

### Analytical validation

Method validation was performed in accordance with the International Council for Harmonisation Q2(R1) guide, considering the intended use of the procedures as rapid semiquantitative screening tests ^22^. Selectivity, system and method linearity, visual detection limit, precision, accuracy expressed as bias, and robustness were evaluated.

Selectivity was evaluated by comparing the Rf values of standard solutions, commercial samples, spiked samples, and samples subjected to stress conditions. The method was considered selective when no relevant interferences were observed and the relative difference between the Rf of the sample and the Rf of the standard was less than or equal to 5%. In the presence of more than one API or potential interferents, a chromatographic resolution equal to or greater than 1.0 was required.

For system linearity, standard solutions were prepared at five concentration levels equivalent to 60%, 80%, 100%, 120%, and 140% of the declared content, with the purpose of verifying the proportionality of the densitometric response over a wide concentration range. For method linearity, spiked samples were prepared at three levels: 80%, 100%, and 120%, because these levels correspond to the expected operating range for the semi-quantitative interpretation of the kit. Each concentration was applied in duplicate on TLC plates and the procedure was repeated three times. The response was evaluated using the densitometric area of the eluted spot. A correlation coefficient r > 0.99 was considered acceptable.

The detection limit was defined as the smallest amount of the API visually detectable by spot size and intensity under the established chromatographic conditions. For this purpose, solutions equivalent to 20% and 40% of the declared content were prepared, applied in duplicate, and evaluated in three complete replicates. A formal quantification limit was not determined because the method was designed for semi-quantitative screening and not for confirmatory quantification.

Precision was evaluated as repeatability and intermediate precision, expressed as the percentage coefficient of variation of the Rf values. Repeatability was evaluated by the same analyst on the same day, using standard solutions and samples spiked at 80%, 100%, and 120%, applied in duplicate on three independent plates. Intermediate precision was evaluated by three analysts, each with three complete replicates of the procedure. The acceptance criteria were a coefficient of variation (CV%) < 3.0% for repeatability and CV% < 5.0% for intermediate precision.

Accuracy was evaluated as bias, comparing the results obtained by TLC-densitometry with those obtained by HPLC at the CNCC of the INS. Bias was analyzed using a 90% bilateral confidence interval, with an acceptance criterion of ±3.00%, a value considered sufficient to guarantee analytically acceptable agreement with the HPLC reference method for the intended use of the procedure; even relative biases less than 5% are considered very demanding when evaluating this parameter.

Robustness was evaluated using a 2^k-p fractional factorial design, considering five factors: mobile phase reagent 1 volume, mobile phase reagent 2 volume, development chamber geometry, development distance, and analyst ^23^. The two components of the mobile phase varied according to the method evaluated, in accordance with the chromatographic composition defined for each API or combination. Each factor was evaluated at two levels. The method was considered robust when deliberate variations did not produce significant changes in Rf values, defined as deviations less than two times the standard deviation observed in intermediate precision.

### Hampi Tariki Kit

The Hampi Tariki kit was designed as a portable set of materials to perform the validated TLC methods under screening conditions. It includes chromatographic plates, developing chambers, capillaries for application, reference materials, an operating manual, and two portable UV light lamps for field reading: one of 254 nm and another of 366 nm.

For the transfer of solvents to fieldwork, the kit uses glass vials, selected to reduce the risk of interaction between organic solvents and packaging materials. The vials must be kept closed, protected from direct light, and under conditions that minimize evaporation or contamination during transport and the analytical workday.

The kit's design is aimed at a short screening session. In the intended application, sample preparation, chromatographic development, drying, visualization, and interpretation are performed in approximately two hours. Therefore, the stability considered in this study corresponds to operational stability during the analytical procedure, not to prolonged storage of prepared solutions.

### Ethical considerations

The study did not involve human beings, experimental animals, or individual clinical information. The analyzed samples corresponded to commercial drugs used exclusively for the development and analytical validation of semiquantitative screening methods. Due to the nature of the study, informed consent was not required. The project was approved by the Institutional Research Ethics Committee of the National Institute of Health (code EI-037-15).

## RESULTS

Eight TLC methods were developed and validated for the semi-quantitative screening of APIs in solid oral dosage forms. The optimal chromatographic conditions are presented in Table 1, including API concentration, mobile phase, detection system, Rf value, and resolution between APIs in multicomponent formulations. The Rf values were within the pre-established range of 0.20 to 0.85 and allowed the identification of the evaluated APIs. In multicomponent combinations, the chromatographic resolution was equal to or greater than 1.0 under the validated conditions.

**Table 1:** Optimal chromatographic conditions of the thin-layer chromatography (TLC) methods for the active pharmaceutical ingredients (APIs) evaluated.

Evaluated APIs	Concentration of API	Mobile phase	Detection method	Rf Value	Resolution between APIs
Aciclovir	200, 400 and 800 mg	Ethyl acetate/methanol/25% ammonia solution (9/11/3 mL)	UV 254 nm	0.56	—
Dicloxacillin	250 and 500 mg	Ethyl acetate/water/glacial acetic acid (15/2/3 mL)	UV 254 nm; iodine vapors	0.70	—
Dimenhydrinate	50 mg	Toluene/ethyl acetate/glacial acetic acid/methanol (8/10/1.5/1 mL)	UV 254 nm	0.60	—
Ibuprofen	200, 400, 600 and 800 mg	Ethyl acetate/methanol/25% ammonia solution (18/4/2 mL)	UV 254 nm	0.28	—
Diclofenac sodium	25, 50 and 100 mg	Toluene/ethyl acetate/glacial acetic acid (9/10/1 mL)	UV 254 nm; iodine vapors	0.61	—
Diclofenac sodium/paracetamol	50/500 mg	Toluene/ethyl acetate/glacial acetic acid (9/10/1 mL)	UV 254 nm; iodine vapors	DCF: 0.62; PCM: 0.33	3.5
Norfloxacin/phenazopyridine hydrochloride	400/100 and 400/50 mg	Ethyl acetate/methanol/water/25% ammonia solution (10/5/0.6/4.4 mL)	UV 254 nm; iodine vapors	FZP: 0.85; NFX: 0.35	8.3
Chlorphenamine maleate/phenylephrine hydrochloride/paracetamol	2/5/500 and 4/10/500 mg	Ethyl acetate/toluene/methanol/25% ammonia solution (14/2/3/1 mL)	UV 254 nm; UV 366 nm; iodine vapors	CFM: 0.68; PCM: 0.53; FEC: 0.44	1.05; 2.16^a^

DCF: sodium diclofenac; PCM: paracetamol; NFX: norfloxacin; FZP: phenazopyridine hydrochloride; FEC: phenylephrine hydrochloride; CFM: chlorphenamine maleate. In dimenhydrinate, the method was oriented to the main marker diphenhydramine.

a In the chlorphenamine maleate/phenylephrine hydrochloride/paracetamol combination, the resolution values correspond to the FEC-PCM and PCM-CFM order, respectively.

The evaluated matrices corresponded to commercial formulations available in the national market. Four acyclovir matrices, seven dicloxacillin matrices, five dimenhydrinate matrices, ten ibuprofen matrices, six sodium diclofenac matrices, ten sodium diclofenac/paracetamol matrices, eight norfloxacin/phenazopyridine hydrochloride matrices, and seven chlorphenamine maleate/phenylephrine hydrochloride/paracetamol matrices were included.

During optimization, diluents were selected that maintained the chromatographic behavior of the samples during the assay execution. Considering that the complete application of rapid methodologies takes approximately two hours, the prepared solutions showed sufficient operational stability for screening within that working window. No visible changes were observed that would impede the reading of Rf, the comparison of spot intensity, or the semiquantitative interpretation during the analytical procedure.

The preparation of stock solutions was adjusted to the concentration of the API declared in each drug, with the purpose of obtaining comparable intermediate solutions among commercial presentations. From these stock solutions, working solutions at 80%, 100%, and 120% were prepared, used as semi-quantitative references for visual and densitometric reading of the spots.

In some methods, the diluent of the working solution was different from that used in the stock solution, as occurred with acyclovir, whose stock solution was prepared in 0.1 M NaOH and its working solutions were prepared with methanol. Preparation conditions are summarized in table 2, while the specific dilutions for each working level are presented in supplementary table 1.

**Table 2:** Preparation of stock solutions for samples and reference materials in the methods by thin-layer chromatography (TLC).

APIs evaluated	Drug concentration	Preparation of the stock solution	Final concentration of the stock solution	Diluent
Aciclovir	200 mg	Crush 1 tablet and dissolve with 40 mL of diluent	5 mg/mL	0.1 M NaOH in water
	400 mg	Crush 1 tablet and dissolve with 80 mL of diluent	5 mg/mL	NaOH 0.1 M in water
	800 mg	Crush 1 tablet and dissolve with 160 mL of diluent	5 mg/mL	NaOH 0.1 M in water
Dicloxacillin, as sodium salt	250 mg	Open 1 capsule and dissolve with 12.5 mL of diluent	20 mg/mL	Methanol
	500 mg	Open 1 capsule and dissolve with 25 mL of diluent	20 mg/mL	Methanol
Dimenhydrinate	50 mg	Crush 1 tablet or open 1 capsule and dissolve with 10 mL of diluent	5 mg/mL	90% aqueous methanol
Ibuprofen	200 mg	Crush 1 tablet or open 1 capsule and dissolve with 20 mL of diluent	10 mg/mL	Methanol
	400 mg	Crush 1 tablet or open 1 capsule and dissolve with 40 mL of diluent	10 mg/mL	Methanol
	600 mg	Crush 1 tablet or open 1 capsule and dissolve with 60 mL of diluent	10 mg/mL	Methanol
	800 mg	Crush 1 tablet or open 1 capsule and dissolve with 80 mL of diluent	10 mg/mL	Methanol
Diclofenac sodium	25 mg	Crush 1 tablet or open 1 capsule and dissolve with 12.5 mL of diluent	2 mg/mL	Methanol
	50 mg	Crush 1 tablet or open 1 capsule and dissolve with 25 mL of diluent	2 mg/mL	Methanol
	100 mg	Crush 1 tablet or open 1 capsule and dissolve with 50 mL of diluent	2 mg/mL	Methanol
Diclofenac sodium/paracetamol	50/500 mg	Crush 1 tablet and dissolve with 25 mL of diluent	DCF: 2 mg/mL; PCM: 20 mg/mL	Methanol
Norfloxacin/phenazopyridine hydrochloride	400/100 mg	Crush 1 tablet or open 1 capsule and dissolve with 80 mL of diluent	NFX: 5 mg/mL; FZP: 1.25 mg/mL	Acidified water
	400/50 mg	Crush 1 tablet or open 1 capsule and dissolve with 80 mL of diluent	NFX: 5 mg/mL; FZP: 0.625 mg/mL	Acidified Water
Chlorphenamine maleate/phenylephrine hydrochloride/paracetamol	2/5/500 mg	Crush 2 tablets, open 2 capsules or dissolve 2 sachets with 10 mL of diluent	CFM: 0.4 mg/mL; FEC: 1 mg/mL; PCM: 100 mg/mL	Methanol
	4/10/500 mg	Crush 2 tablets, open 2 capsules or dissolve 2 sachets with 10 mL of diluent	CFM: 0.8 mg/mL; FEC: 2 mg/mL; PCM: 100 mg/mL	Methanol

DCF: sodium diclofenac; PCM: paracetamol; NFX: norfloxacin; FZP: phenazopyridine hydrochloride; CFM: chlorphenamine maleate; FEC: phenylephrine hydrochloride.

Acidified water: mixture of 2 mL of glacial acetic acid with 78 mL of water.

In the case of dimenhydrinate, the method was oriented to the main marker diphenhydramine.

The stock solution was used as an intermediate solution to prepare the working solutions at 80%, 100%, and 120%. The diluent indicated in this table corresponds to the preparation of the stock solution; the diluents and volumes used for the working solutions are detailed in Supplementary Table 1. For acyclovir, the stock solution was prepared in 0.1 M NaOH, while the working solutions were prepared with methanol. For ibuprofen, the 10 mg/mL stock solution was used as an intermediate to prepare the 100% working solution of 8 mg/mL, defined as the operative reference for semiquantitative comparison.

In supplementary figure 1, for demonstrative purposes of the method's chromatographic performance, a TLC plate of the phenylephrine hydrochloride/paracetamol/chlorphenamine maleate combination is shown, used to illustrate the chromatographic separation of the three APIs under UV light at 254 nm and 366 nm. On this plate, the lanes labeled as 80% and 100% correspond to semiquantitative comparison references, and the sample lanes allow observing the presence or absence of the spots corresponding to the evaluated ingredients.

Analytical validation is summarized in table 3. All methods met the pre-established linearity criteria, with correlation coefficients greater than 0.99 for the system and the method. Specificity/selectivity was adequate, as the relative difference between the sample Rf and the standard Rf remained within the ≤5% criterion, without relevant interferences from the diluent, excipients, or matrix components; in multicomponent formulations, chromatographic resolution was ≥1.0 under the validated conditions. Precision, evaluated under repeatability and intermediate conditions, showed coefficients of variation within the acceptance limits; accuracy, expressed as bias against HPLC, remained within the ±3.00% range, and robustness demonstrated that deliberate variations of the evaluated factors did not produce significant changes in Rf values. In dimenhydrinate, the interpretation was oriented towards diphenhydramine as the main marker.

**Table 3:** Analytical validation parameters of thin-layer chromatography (TLC) methods.

Evaluated API	Specificity/selectivity	Linearity system r	Linearity method r	Visual detection limit (mg/spot) ^a^	Repeatability CV%	Intermediate precision CV%	Bias, CI 90%	Robustness
CVA	Complies; sample/standard Rf ≤5%	0.9958	0.9966	0.050	1.53	4.89	-0.57 to 0.37	0.0465
DCX	Complies; impending degradation under acid hydrolysis	0.9917	0.9966	6.400	1.68	3.63	0.01 to 0.51	0.0547
DMH	Serves for diphenhydramine as a marker	0.9966	0.9979	0.800	1.38	1.51	-1.15 to 0.37	0.0156
IBP	Meets; Rf sample/standard ≤5%	0.9954	0.9995	3.200	1.77	4.86	-1.09 to 0.58	0.0199
DCF	Complies; Rf sample/standard ≤5%	0.9942	0.9943	0.150	1.62	1.52	-0.25 to 0.83	0.0465
DCF in DCF/PCM	Complies; resolution ≥1.0	0.9960	0.9984	0.150	1.20	4.45	0.00 to 1.19	0.0191
PCM in DCF/PCM	Meets; resolution ≥1.0	0.9947	0.9970	0.750	1.86	3.77	-0.52 to 0.45	0.0414
FZP in FZP/NFX	Meets; resolution ≥1.0	0.9972	0.9960	0.250	1.74	3.21	0.38 to 0.78	0.0190
NFX in FZP/NFX	Meets; resolution ≥1.0	0.9968	0.9977	0.125	1.87	4.92	-0.17 to 0.61	0.0263
CFM in CFM/FEC/PCM	Meets; resolution ≥1.0	0.9950	0.9965	0.120	2.33	2.40	-0.83 to 0.08	0.0517
FEC in CFM/FEC/PCM	Complies; resolution ≥1.0	0.9960	0.9939	0.030	2.89	3.11	-0.30 to 0.58	0.0290
PCM in CFM/FEC/PCM	Complies; resolution ≥1.0	0.9968	0.9945	0.018	2.72	2.76	-0.02 to 0.94	0.0198

r: correlation coefficient; CV: coefficient of variation; CI: confidence interval; ACV: acyclovir; DCX: dicloxacillin; DMH: dimenhydrinate; IBP: ibuprofen; DCF: diclofenac sodium; PCM: paracetamol; FZP: phenazopyridine hydrochloride; NFX: norfloxacin; CFM: chlorphenamine maleate; FEC: phenylephrine hydrochloride.

a The reported parameter corresponds to the visual detection limit, not to the formal quantification limit.

The Hampi Tariki portable kit is shown in Figure 1. The design integrates the necessary materials to execute the methods in a short screening day: chromatographic plates, developing chambers, capillaries, reference materials, operating manual, glass vials for solvent transport, and two portable UV light lamps of 254 nm and 366 nm. This configuration allows for reading the plates according to the detection system defined for each API.

**Figure 1:**
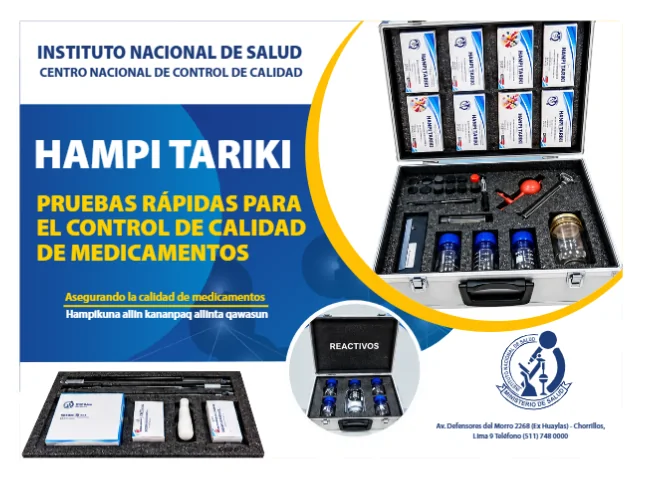
Interior design of the Hampi Tariki portable kit.

The kit contains chromatographic plates, development chambers, glass vials for solvent transport, reference materials, capillaries or applicators, an operating manual, and two UV light lamps for field reading, one of 254 nm and one of 366 nm.

## DISCUSSION

In this study, eight TLC methods were developed and validated for the semi-quantitative screening of APIs in tablets and capsules marketed in Peru. The methods allowed differentiating levels equivalent to 80%, 100%, and 120% of the declared content through visual and densitometric comparison of the Rf, size, and intensity of the spots. This approach is consistent with the use of TLC as a screening tool, rather than as a confirmatory quantification method ^8^^-^^13^.

The specificity/selectivity results support the use of the methods as preliminary screening tests, since the APIs could be identified in commercial matrices without relevant interferences and with Rf differences within the acceptance criterion. This point is important because brand-name and generic products from different manufacturers were evaluated, with possible variations in excipients. In multicomponent drugs, chromatographic separation allowed differentiating the APIs under the validated conditions; however, these results should not be extrapolated to unevaluated combinations. In dimenhydrinate, the interpretation was limited to diphenhydramine as the main marker, because 8-chlorotheophylline presented a low Rf and was not validated as an independent marker.

Linearity was satisfactory for all evaluated APIs, with correlation coefficients greater than 0.99, which evidences proportionality between the concentration and the densitometric area of the spot within the working range. This result supports the method's usefulness for differentiating semiquantitative levels, especially when the objective is to identify marked deviations with respect to the declared content.

Precision was adequate under repeatability and intermediate precision conditions, with coefficients of variation within the predefined limits. The participation of three analysts in the intermediate precision strengthens the applicability of the method, given that a screening test must maintain acceptable performance when performed by different operators. Furthermore, the evaluation of robustness showed that the deliberate variations evaluated did not produce significant changes in Rf values, which suggests stability of the chromatographic behavior under small variations of the procedure.

Accuracy, expressed as bias against HPLC, remained within the defined acceptance range. This comparison should be understood as a preliminary evaluation of analytical agreement, given that the methods correspond to semiquantitative tests. In this sense, their application is relevant as a complementary tool for the rapid verification of drug quality, therefore, the methods should not be presented as substitutes for confirmatory instrumental techniques ^10^^-^^13^.

The usefulness of TLC for drug screening has been previously documented. Roy et al. demonstrated that simple TLC methods can be employed as initial screening tests, although they do not replace official pharmacopoeial methods ^8^. Similarly, studies with GPHF-Minilab have shown that this technology can identify products with absence of API, incorrect API, or markedly low content, especially in resource-limited settings ^9^^,^^10^^,^^13^^,^^14^. However, the literature also points out that these methods have less capacity to detect moderate content deviations or dissolution problems, so suspicious samples require confirmation using pharmacopoeial or instrumental methods ^10^^-^^15^.

The selection of the evaluated APIs is related to national backgrounds of drug falsification in oral solid forms and in frequently used therapeutic groups, including analgesics, anti-inflammatories, anti-infectives, and symptomatic combinations ^1^^,^^2^. In that context, the developed methods cover a set of products relevant for preliminary health surveillance in the country, although their application must be limited to the evaluated matrices and combinations.

A relevant observation for the operational use of the kit is the stability of the solutions during the assay. In this study, the selection of the diluent considered not only the solubility and recovery of the analyte, but also the chromatographic behavior during the execution of the procedure. Given that the complete application of rapid methodologies takes approximately two hours, the results support the operational stability of the solutions during that analytical window. This evidence should not be interpreted as a formal extended stability study, nor does it allow for establishing extended storage times for prepared solutions.

The use of glass vials for solvent transport constitutes an important operational feature of the kit. This choice reduces the risk of incompatibility between the solvents and packaging materials, a valid concern when toluene or ethyl acetate are used. However, for routine field implementation, it will be necessary to document simple controls before use, such as closure verification, absence of anomalous coloration, absence of particles, and absence of visible evaporation. These controls do not replace a formal compatibility study, but they help reduce operational risks during short screening sessions.

The portability of the kit and the relative simplicity of TLC can favor its use as a complementary decentralized surveillance tool. Unlike a generic description of instrumental reading, the Hampi Tariki kit incorporates two independent UV lamps, one of 254 nm and the other of 366 nm, which allows field readings to be performed according to the detection system established for each API. Furthermore, development with iodine vapors represents a useful alternative when UV visualization is not sufficient or when a complementary detection system is required ^13^^,^^14^.

The strengths of the study include the development of methods for several APIs of health interest, the inclusion of single-component and multi-component formulations, the evaluation of 57 commercial matrices available in the national market, the use of previously defined acceptance criteria, and the comparison of bias against HPLC. Furthermore, the study stems from a documented health need in Peru, where counterfeiting primarily affects high-circulation oral solid medications ^1^^,^^2^. In terms of public health, the Hampi Tariki kit could contribute to decentralized health surveillance by prioritizing suspicious samples for confirmation in official laboratories. Its potential value lies in offering initial analytical guidance in scenarios where it is necessary to decide which samples should be sent with higher priority for instrumental confirmation. Regarding cost, the kit could represent an operationally accessible alternative compared to instrumental technologies or imported commercial kits. However, this statement should be interpreted as a potential advantage, given that the present study did not include a formal economic evaluation nor a comparative cost analysis against other screening technologies ^(^^24^.

This study also presents limitations that must be acknowledged to adequately contextualize the scope of the results. The validation was carried out with reference standards and commercial medicines previously verified as compliant, without including actual falsified samples, seized products, or adulterated formulations. Therefore, the findings do not allow for direct estimation of the kit's performance against falsified medicines present in the market. Another relevant limitation is the absence of evaluation under field conditions, where factors such as environmental variations, available lighting, operator experience, and sample referral logistics can influence performance. Likewise, the documented stability corresponds to the approximate two-hour operational window during method application, without considering scenarios of prolonged storage of prepared solutions.

In conclusion, eight thin-layer chromatography methods integrated into the Hampi Tariki kit were developed and validated for the semi-quantitative screening of APIs in oral solid medications; under controlled laboratory conditions, the methods showed adequate analytical performance and allowed for the differentiation of levels equivalent to 80%, 100%, and 120% of the declared content, which constitute a preliminary screening proposal to identify suspicious samples and prioritize their confirmation using HPLC or other official methods, without replacing confirmatory pharmacopeial procedures.
