# Supplementary material for: Hampi Tariki kit: thin-layer chromatography for semiquantitative screening of solid oral medicines in Peru
Source: Rev Peru Med Exp Salud Publica. 2026 Aug 28;43(3):311–20. doi: 10.17843/rpmesp.2026.433.15446 (PMC13718568; doi:10.17843/rpmesp.2026.433.15446)
Supplement: Supplementary file 1 [file rpmesp-43-03-15446-s001-es.docx]

**Tabla suplementaria 1. Preparación de soluciones de trabajo al 80%, 100% y 120% para la interpretación semicuantitativa por CCF**

| **IFAs evaluados** | **Solución de trabajo al 100%** | **Solución de trabajo al 80%** | **Solución de trabajo al 120%** |
| --- | --- | --- | --- |
| Aciclovir | 0,25 mg/mL: 1 mL de SM + 19 mL de metanol | 0,20 mg/mL: 0,8 mL de SM + 19,2 mL de metanol | 0,30 mg/mL: 1,2 mL de SM + 18,8 mL de metanol |
| Dicloxacilina | 8 mg/mL: 4 mL de SM + 6 mL de metanol | 6,4 mg/mL: 3,2 mL de SM + 6,8 mL de metanol | 9,6 mg/mL: 4,8 mL de SM + 5,2 mL de metanol |
| Dimenhidrinato | 4 mg/mL: 8 mL de SM + 2 mL de metanol acuoso al 90% | 3,2 mg/mL: 6,4 mL de SM + 3,6 mL de metanol acuoso al 90% | 4,8 mg/mL: 9,6 mL de SM + 0,4 mL de metanol acuoso al 90% |
| Ibuprofeno | 8 mg/mL: 4 mL de SM + 1 mL de metanol | 6,4 mg/mL: 3,2 mL de SM + 1,8 mL de metanol | 9,6 mg/mL: 4,8 mL de SM + 0,2 mL de metanol |
| Diclofenaco sódico | 0,375 mg/mL: 1,5 mL de SM + 6,5 mL de metanol | 0,30 mg/mL: 1,2 mL de SM + 6,8 mL de metanol | 0,45 mg/mL: 1,8 mL de SM + 6,2 mL de metanol |
| Diclofenaco sódico/paracetamol | DCF: 0,375 mg/mL; PCM: 3,75 mg/mL. Preparar con 1,5 mL de SM + 6,5 mL de metanol | DCF: 0,30 mg/mL; PCM: 3,0 mg/mL. Preparar con 1,2 mL de SM + 6,8 mL de metanol | DCF: 0,45 mg/mL; PCM: 4,5 mg/mL. Preparar con 1,8 mL de SM + 6,2 mL de metanol |
| Norfloxacino/fenazopiridina clorhidrato 400/100 mg | NFX: 1,25 mg/mL; FZP: 0,3125 mg/mL. Preparar con 2 mL de SM + 6 mL de metanol | NFX: 1 mg/mL; FZP: 0,25 mg/mL. Preparar con 1,6 mL de SM + 6,4 mL de metanol | NFX: 1,5 mg/mL; FZP: 0,375 mg/mL. Preparar con 2,4 mL de SM + 5,6 mL de metanol |
| Norfloxacino/fenazopiridina clorhidrato 400/50 mg | NFX: 1,25 mg/mL, con 2 mL de SM + 6 mL de metanol.  FZP: 0,3125 mg/mL, con 4 mL de SM + 4 mL de metanol | NFX: 1 mg/mL, con 1,6 mL de SM + 6,4 mL de metanol.  FZP: 0,25 mg/mL, con 3,2 mL de SM + 4,8 mL de metanol | NFX: 1,5 mg/mL, con 2,4 mL de SM + 5,6 mL de metanol.  FZP: 0,375 mg/mL, con 4,8 mL de SM + 3,2 mL de metanol |
| Clorfenamina maleato/fenilefrina clorhidrato/paracetamol 2/5/500 mg | CFM: 0,3 mg/mL; FEC: 0,75 mg/mL; PCM: 75 mg/mL. Preparar con 3 mL de SM + 1 mL de metanol | CFM: 0,24 mg/mL; FEC: 0,6 mg/mL; PCM: 60 mg/mL. Preparar con 2,4 mL de SM + 1,6 mL de metanol | CFM: 0,36 mg/mL; FEC: 0,9 mg/mL; PCM: 90 mg/mL. Preparar con 3,6 mL de SM + 0,4 mL de metanol |
| Alternativa para FEC/PCM en formulación 2/5/500 mg | FEC: 0,15 mg/mL; PCM: 15 mg/mL. Preparar con 1,5 mL de SM + 8,5 mL de metanol | FEC: 0,12 mg/mL; PCM: 12 mg/mL. Preparar con 1,2 mL de SM + 8,8 mL de metanol | FEC: 0,18 mg/mL; PCM: 18 mg/mL. Preparar con 1,8 mL de SM + 8,2 mL de metanol |
| Clorfenamina maleato/fenilefrina clorhidrato/paracetamol 4/10/500 mg | CFM: 0,3 mg/mL; FEC: 0,75 mg/mL, con 1,5 mL de SM + 2,5 mL de metanol.  PCM: 75 mg/mL, con 3 mL de SM + 1 mL de metanol | CFM: 0,24 mg/mL; FEC: 0,6 mg/mL, con 1,2 mL de SM + 2,8 mL de metanol.  PCM: 60 mg/mL, con 2,4 mL de SM + 1,6 mL de metanol | CFM: 0,36 mg/mL; FEC: 0,9 mg/mL, con 1,8 mL de SM + 2,2 mL de metanol.  PCM: 90 mg/mL, con 3,6 mL de SM + 0,4 mL de metanol |
| Alternativa para FEC/PCM en formulación 4/10/500 mg | FEC: 0,15 mg/mL, con 0,75 mL de SM + 9,25 mL de metanol.  PCM: 15 mg/mL, con 1,5 mL de SM + 8,5 mL de metanol | FEC: 0,12 mg/mL, con 0,6 mL de SM + 9,4 mL de metanol.  PCM: 12 mg/mL, con 1,2 mL de SM + 8,8 mL de metanol | FEC: 0,18 mg/mL, con 0,9 mL de SM + 9,1 mL de metanol.  PCM: 18 mg/mL, con 1,8 mL de SM + 8,2 mL de metanol |

SM: solución madre; DCF: diclofenaco sódico; PCM: paracetamol; NFX: norfloxacino; FZP: fenazopiridina clorhidrato; CFM: clorfenamina maleato; FEC: fenilefrina clorhidrato.
Las soluciones al 80%, 100% y 120% se usaron como referencias visuales y densitométricas para la interpretación semicuantitativa del contenido declarado.

La solución madre fue empleada como solución intermedia; por ello, su concentración no necesariamente coincide con la concentración de trabajo al 100%. En la presentación NFX/FZP 400/100 mg, las soluciones de trabajo de ambos IFAs se prepararon a partir de una dilución común de la solución madre; en la presentación NFX/FZP 400/50 mg, se prepararon diluciones diferenciadas para NFX y FZP, con el fin de obtener concentraciones de trabajo comparables para la lectura semicuantitativa. Las alternativas para FEC y PCM se emplean cuando se requiere mejorar la visualización de las manchas cromatográficas.

**100%	M_1_	80%	M_2_**

**100%	M_1_	80%	M_2_**

**UV 254 nm**

**FEC**

**CFM**

**PCM**

**Figura suplementaria 1.** Placa de CCF de la combinación fenilefrina clorhidrato/paracetamol/clorfenamina maleato. Se muestra la separación cromatográfica de los tres IFAs bajo luz UV de 254 nm y 366 nm. La interpretación semicuantitativa se realizó mediante comparación visual de las manchas de las muestras con las referencias equivalentes al 80% y 100% del contenido declarado. Los carriles de muestra se presentan con fines demostrativos de la separación cromatográfica y no corresponden a la identificación de un producto comercial falsificado.
