# Supplementary material for: Hampi Tariki kit: thin-layer chromatography for semiquantitative screening of solid oral medicines in Peru
Source: Rev Peru Med Exp Salud Publica. 2026 Aug 28;43(3):311–20. doi: 10.17843/rpmesp.2026.433.15446 (PMC13718568; doi:10.17843/rpmesp.2026.433.15446)
Supplement: Translation

# Translation: Kit Hampi Tariki: cromatografía en capa fina para el tamizaje semicuantitativo de medicamentos sólidos orales en Perú

**Authors:** Gladys M. Mónico-Rosales, Fredy R. Mostacero-Rodríguez, B. Rosa Quispe-Paitán, E. Javier Rodríguez-Calzado, Juan J. Ortiz-Bernaola, Alejandro Nuñez-Galindo

**Affiliations:** 1 Centro Nacional de Control de Calidad, Instituto Nacional de Salud, Lima, Perú

**Keywords:** Medicamentos Falsificados, Cromatografía en Capa Delgada, Control de Calidad, Vigilancia Sanitaria, Tecnología Farmacéutica

## Abstract

**Objetivos.:**

Desarrollar y validar métodos rápidos por cromatografía en capa fina (CCF) integrados en el kit Hampi Tariki para el tamizaje semicuantitativo de ingredientes farmacéuticos activos (IFAs) en medicamentos sólidos orales comercializados en el Perú

**Materiales y métodos.:**

Se desarrollaron y validaron ocho métodos por CCF para tabletas y cápsulas con aciclovir, dicloxacilina, dimenhidrinato, ibuprofeno, diclofenaco sódico, paracetamol, norfloxacino, fenazopiridina clorhidrato, clorfenamina maleato y fenilefrina clorhidrato. Se evaluaron 57 muestras comerciales disponibles en el mercado nacional, procedentes de diferentes laboratorios fabricantes, incluyendo productos de marca y genéricos previamente verificados como conformes por métodos oficiales en el Centro Nacional de Control de Calidad (CNCC) del Instituto Nacional de Salud (INS). Se utilizaron placas de gel de sílice 60 F254, estándares de referencia y disolventes de grado analítico. La validación se realizó según la guía ICH Q2(R1), evaluando selectividad, linealidad, precisión, exactitud, límite de detección visual y robustez.

**Resultados.:**

Los métodos permitieron diferenciar visual y densitométricamente niveles equivalentes al 80%, 100% y 120% del contenido declarado. Los valores del factor de retención (Rf) se ubicaron entre 0,20 y 0,85, y las formulaciones multicomponente presentaron resolución cromatográfica ≥1,0. Los coeficientes de correlación fueron superiores a 0,99 para la linealidad del sistema y del método; la repetibilidad presentó coeficientes de variación menores a 3,0%, la precisión intermedia menores a 5,0%, y el sesgo frente a cromatografía líquida de alta eficiencia (HPLC) se mantuvo dentro del intervalo de ±3,00%. Las variaciones deliberadas evaluadas en robustez no produjeron cambios significativos en los valores de Rf.

**Conclusiones.:**

Los métodos desarrollados demostraron desempeño analítico adecuado como pruebas rápidas de tamizaje semicuantitativo. Integrados en el kit Hampi Tariki, constituyen una propuesta con potencial para apoyar la identificación preliminar de muestras sospechosas y priorizar su confirmación mediante HPLC u otros métodos oficiales.

## INTRODUCCIÓN

Los medicamentos falsificados representan un problema relevante de salud pública, debido a que pueden contener ingredientes incorrectos, ausencia total o parcial del ingrediente farmacéutico activo (IFA), concentraciones diferentes a las declaradas, envases falsificados o información fraudulenta sobre su identidad, composición u origen ^1^. Estos productos pueden ocasionar fallos terapéuticos, eventos adversos, pérdida de confianza en los sistemas sanitarios y, en el caso de los antiinfecciosos, contribuir a la resistencia antimicrobiana ^1^^-^^3^.

En el Perú, la falsificación de medicamentos constituye un problema documentado. Medina *et al.* Analizaron las alertas emitidas por la Dirección General de Medicamentos, Insumos y Drogas (DIGEMID) entre 1997 y 2014, e identificaron 1738 casos de medicamentos falsificados; el 58,1% estuvo asociado con establecimientos farmacéuticos y el 20,1% con establecimientos comerciales no farmacéuticos ^1^. Además, las tabletas fueron la forma farmacéutica más frecuente y los analgésicos y antibacterianos sistémicos estuvieron entre los grupos terapéuticos más afectados ^1^. De manera más reciente, Gutiérrez-García *et al*. Analizaron alertas nacionales de falsificación emitidas entre 2013 y 2024, e identificaron 1247 productos farmacéuticos falsificados, predominantemente medicamentos de marca comercial, formas sólidas orales y productos de uso frecuente como analgésicos, antipiréticos, antiinflamatorios, vitaminas y antibacterianos ^2^.

La vigilancia sanitaria de estos productos requiere métodos confirmatorios confiables, como la cromatografía líquida de alta eficiencia (HPLC, por sus siglas en inglés) u otros procedimientos farmacopeicos. Sin embargo, estos análisis demandan laboratorios equipados, personal especializado, reactivos de grado analítico y tiempos de respuesta que pueden limitar su aplicación inmediata en intervenciones descentralizadas ^4^^-^^7^. Por ello, las tecnologías de tamizaje rápido se han propuesto como herramientas complementarias para orientar decisiones iniciales y priorizar muestras sospechosas, especialmente en escenarios donde el acceso inmediato a un laboratorio instrumental es limitado ^5^^-^^7^.

La cromatografía en capa fina (CCF), utilizada en iniciativas como el Global Pharma Health Fund Minilab (GPHF-Minilab), permite evaluar la identidad del IFA y realizar una estimación semicuantitativa mediante la comparación del tamaño, color, intensidad y factor de retención (Rf) de las manchas de la muestra frente a materiales de referencia ^8^^-^^13^. Esta tecnología de bajo costo ha mostrado utilidad para reconocer productos con ausencia de IFA, ingrediente incorrecto o contenido marcadamente bajo; sin embargo, no reemplaza a los métodos confirmatorios oficiales ni permite, por sí sola, establecer conformidad farmacopeica completa ^9^^-^^15^.

En este contexto, resulta pertinente disponer de métodos rápidos por CCF adaptados a medicamentos sólidos orales de interés para la vigilancia sanitaria en el Perú. Por ello, el objetivo del estudio fue desarrollar y validar métodos analíticos rápidos por CCF para el tamizaje semicuantitativo de IFAs en medicamentos sólidos orales integrados en el kit Hampi Tariki.

**MENSAJES CLAVE**

Motivación del estudio. Los medicamentos falsificados constituyen un problema de salud pública en el Perú, pues pueden causar fallas terapéuticas, reacciones adversas y resistencia antimicrobiana. La vigilancia sanitaria requiere herramientas rápidas, portátiles y accesibles.

Principales hallazgos. Se desarrollaron y validaron ocho métodos de cromatografía en capa fina, integrados en el kit Hampi Tariki, para detectar ingredientes farmacéuticos activos en medicamentos sólidos orales. Los métodos diferenciaron contenidos equivalentes al 80%, 100% y 120% de lo declarado. Los métodos validados demostraron su desempeño analítico conforme a directrices internacionales.

Implicancias en salud pública. El kit permite identificar preliminarmente muestras sospechosas y priorizar su análisis confirmatorio, como complemento y no sustituto de los métodos farmacopeicos oficiales..

## MATERIALES Y MÉTODOS

Se realizó un estudio analítico de desarrollo y validación de métodos rápidos por CCF para el tamizaje semicuantitativo de IFAs en medicamentos sólidos orales.

### Muestras farmacéuticas

La población de estudio estuvo constituida por medicamentos registrados en DIGEMID y comercializados en el Perú, en formas farmacéuticas sólidas de administración oral: tabletas y cápsulas. Se incluyeron formulaciones monocomponente de aciclovir, dicloxacilina, dimenhidrinato, ibuprofeno y diclofenaco sódico, así como combinaciones de diclofenaco sódico/paracetamol, norfloxacino/fenazopiridina clorhidrato y clorfenamina maleato/fenilefrina clorhidrato/paracetamol.

Las muestras correspondieron a medicamentos de marca comercial y genéricos, procedentes de diferentes laboratorios fabricantes, concentraciones y formulaciones comerciales disponibles en el mercado nacional. En total, se evaluaron 57 matrices comerciales: aciclovir de cuatro laboratorios fabricantes, dicloxacilina de siete, dimenhidrinato de cinco, ibuprofeno de diez, diclofenaco sódico de seis, diclofenaco sódico/paracetamol de diez, norfloxacino/fenazopiridina clorhidrato de ocho y clorfenamina maleato/fenilefrina clorhidrato/paracetamol de siete.

Todas las muestras utilizadas para la validación fueron previamente evaluadas mediante métodos oficiales de control de calidad, incluyendo HPLC cuando correspondía, y contaban con resultados conformes. Por tanto, las muestras empleadas no correspondieron a medicamentos falsificados reales ni a productos incautados, sino a productos conformes utilizados para el desarrollo, optimización y validación analítica de los métodos.

La selección de los IFAs respondió a antecedentes nacionales que describan la falsificación de medicamentos en formas sólidas orales y en grupos terapéuticos de uso frecuente, como analgésicos, antiinflamatorios, antibacterianos y combinaciones sintomáticas ^1^^,^^2^. Complementariamente, se consideró la disponibilidad de estos productos en el mercado nacional y su pertinencia para vigilancia sanitaria.

### Reactivos y estándares

Se utilizaron estándares de referencia USP (United States Pharmacopeia*,* Estados Unidos) de aciclovir, clorfenamina maleato, diclofenaco sódico, dicloxacilina, dimenhidrinato, fenazopiridina clorhidrato, fenilefrina clorhidrato, ibuprofeno, paracetamol y norfloxacino.

El metanol, acetato de etilo, tolueno y solución de amoniaco al 25% procedió de Merck (Darmstadt, Alemania) y el ácido acético glacial de J.T. Baker (Avantor, Estados Unidos). Se utilizó el sistema purificador Milli-Q Advantage A10 (Millipore, Francia) para producir agua ultrapura. Durante el desarrollo se priorizó el uso de disolventes de menor impacto ambiental relativo, evitando el empleo de benceno, cloroformo y ácido fórmico. Cuando fue analíticamente viable, el cloroformo fue reemplazado por tolueno y el ácido fórmico por ácido acético glacial.

### Desarrollo y optimización de los métodos por CCF

El desarrollo de los métodos se realizó con base en el modelo de ensayos por CCF del GPHF-Minilab y en métodos previamente publicados cuando estuvieron disponibles ^8^^,^^13^^,^^16^^-^^21^. Para los IFAs o combinaciones no cubiertas por la literatura, se evaluaron diferentes mezclas de disolventes considerando la polaridad de los componentes, la lipofilia de los IFAs y la necesidad de obtener valores de Rf adecuados para lectura visual y densitométrica.

Durante el desarrollo metodológico se ensayaron diferentes combinaciones de disolventes para la fase móvil y distintos diluyentes de muestra. La selección final consideró valores de Rf adecuados, manchas definidas, recuperación apropiada del IFA y estabilidad operativa de las soluciones durante el tiempo de ejecución del ensayo. En condiciones de tamizaje rápido, la aplicación completa de las metodologías toma aproximadamente dos horas; dentro de esta ventana analítica, no se observaron cambios visibles que impidieran la lectura del Rf, la comparación de intensidad o la interpretación semicuantitativa. Esta observación corresponde a estabilidad operativa durante el uso del método y no remplaza un estudio formal de estabilidad bajo condiciones controladas de almacenamiento.

Se consideró aceptable un sistema cromatográfico cuando produjo valores de Rf entre 0,20 y 0,85, manchas definidas y, en formulaciones multicomponente, resolución igual o superior a 1,0 entre IFAs o frente a posibles interferentes. En los sistemas multicomponente, la evaluación se limitó a las combinaciones comerciales incluidas en el estudio, por lo que los resultados no deben extrapolarse a otras combinaciones de IFAs sin verificación experimental adicional.

Las placas cromatográficas de gel de sílice 60 F254 con soporte de aluminio, de 5 × 10 cm y 20 × 10 cm, se desarrollaron en cámaras cromatográficas saturadas con papel de filtro. Las soluciones se aplicaron a 1,5 cm del borde inferior de la placa. La distancia de desarrollo de la fase móvil fue de 7,5 cm y el tiempo de saturación seleccionado fue de 15 minutos, luego de evaluar tiempos de 12, 15 y 18 minutos. La visualización se realizó bajo luz ultravioleta (UV) de 254 nm o 366 nm, según el IFA, y mediante revelado con vapores de yodo cuando correspondía al método.

En el caso de dimenhidrinato, el método se orientó al seguimiento cromatográfico de difenhidramina como marcador principal. Aunque el dimenhidrinato corresponde a una sal formada por difenhidramina y 8-cloroteofilina, esta última no fue validada como marcador independiente en el presente estudio. Por ello, el alcance del método debe interpretarse como tamizaje semicuantitativo del componente difenhidramina en formulaciones de dimenhidrinato.

### Criterio de interpretación semicuantitativa

La evaluación semicuantitativa se basó en la comparación del Rf, tamaño, color e intensidad de la mancha de la muestra frente a materiales de referencia preparados a concentraciones equivalentes al 80%, 100% y 120% del contenido declarado del IFA. Para la lectura visual, se consideró que una muestra era compatible con el contenido esperado cuando la mancha presentaba el mismo Rf y una intensidad comparable a la referencia del 100%, sin ser inferior a la referencia del 80% ni mostrar manchas adicionales atribuibles a IFAs no declarados o interferentes relevantes.

Las muestras con manchas de intensidad menor que la referencia del 80%, ausencia de la mancha correspondiente al IFA, Rf no concordante con el estándar, presencia de manchas adicionales no explicadas o separación deficiente fueron consideradas sospechosas y requerirían confirmación mediante métodos oficiales, tales como HPLC u otros procedimientos farmacopeicos. Este criterio no constituye una cuantificación definitiva del contenido del IFA, sino una estimación semicuantitativa orientada al tamizaje preliminar ^8^^-^^13^.

### Validación analítica

La validación de los métodos se realizó conforme a la guía del International Council for Harmonisation Q2(R1), considerando el uso previsto de los procedimientos como pruebas rápidas de tamizaje semicuantitativo ^22^. Se evaluaron selectividad, linealidad del sistema y del método, límite de detección visual, precisión, exactitud expresada como sesgo y robustez.

La selectividad se evaluó comparando los valores de Rf de las soluciones estándar, muestras comerciales, muestras adicionadas y muestras sometidas a condiciones de estrés. El método se consideró selectivo cuando no se observaron interferencias relevantes y la diferencia relativa entre el Rf de la muestra y el Rf del estándar fue menor o igual a 5%. En presencia de más de un IFA o de posibles interferentes, se exigió una resolución cromatográfica igual o superior a 1,0.

Para la linealidad del sistema se prepararon soluciones estándar a cinco niveles de concentración equivalentes al 60%, 80%, 100%, 120% y 140% del contenido declarado, con el propósito de verificar la proporcionalidad de la respuesta densitométrica en un intervalo amplio de concentración. Para la linealidad del método se prepararon muestras adicionadas a tres niveles: 80%, 100% y 120%, debido a que estos niveles corresponden al intervalo operativo previsto para la interpretación semicuantitativa del kit. Cada concentración se aplicó por duplicado en placas de CCF y el procedimiento se repitió tres veces. La respuesta se evaluó mediante el área densitométrica de la mancha eluida. Se consideró aceptable un coeficiente de correlación r > 0,99.

El límite de detección se definió como la menor cantidad del IFA detectable visualmente por tamaño e intensidad de la mancha bajo las condiciones cromatográficas establecidas. Para ello se prepararon soluciones equivalentes al 20% y 40% del contenido declarado, aplicadas por duplicado y evaluadas en tres repeticiones completas. No se determinó un límite de cuantificación formal, debido a que el método fue diseñado para tamizaje semicuantitativo y no para cuantificación confirmatoria.

La precisión se evaluó como repetibilidad y precisión intermedia, expresada como coeficiente de variación porcentual de los valores de Rf. La repetibilidad fue evaluada por un mismo analista en un mismo día, utilizando soluciones estándar y muestras adicionadas al 80%, 100% y 120%, aplicadas por duplicado en tres placas independientes. La precisión intermedia fue evaluada por tres analistas, cada uno con tres réplicas completas del procedimiento. Los criterios de aceptación fueron coeficiente de variación (CV%) < 3,0% para repetibilidad y CV% < 5,0% para precisión intermedia.

La exactitud se evaluó como sesgo, comparando los resultados obtenidos por CCF-densitometría con los obtenidos por HPLC en el CNCC del INS. El sesgo se analizó mediante intervalo de confianza bilateral al 90%, con criterio de aceptación de ±3,00%, valor considerado suficiente para garantizar una concordancia analíticamente aceptable con el método de referencia por HPLC para el uso previsto del procedimiento; inclusive sesgos relativos menores a 5% son considerados muy exigentes al evaluar este parámetro.

La robustez se evaluó mediante un diseño factorial fraccionado 2^k-p, considerando cinco factores: volumen del reactivo 1 de la fase móvil, volumen del reactivo 2 de la fase móvil, geometría de la cámara de desarrollo, distancia de desarrollo y analista ^23^. Los dos componentes de la fase móvil variaron según el método evaluado, de acuerdo con la composición cromatográfica definida para cada IFA o combinación. Cada factor se evaluó en dos niveles. El método se consideró robusto cuando las variaciones deliberadas no produjeron cambios significativos en los valores de Rf, definidos como desviaciones menores a dos veces la desviación estándar observada en la precisión intermedia.

### Kit Hampi Tariki

El kit Hampi Tariki fue diseñado como un conjunto portátil de materiales para ejecutar los métodos de CCF validados en condiciones de tamizaje. Incluye placas cromatográficas, cámaras de desarrollo, capilares para aplicación, materiales de referencia, manual operativo y dos lámparas portátiles de luz UV para lectura en campo: una de 254 nm y otra de 366 nm.

Para el traslado de los disolventes al trabajo de campo, el kit utiliza frascos de vidrio, seleccionados para reducir el riesgo de interacción entre los disolventes orgánicos y los materiales de envase. Los frascos deben mantenerse cerrados, protegidos de la luz directa y bajo condiciones que minimicen evaporación o contaminación durante el transporte y la jornada analítica.

El diseño del kit está orientado a una jornada corta de tamizaje. En la aplicación prevista, la preparación de muestras, desarrollo cromatográfico, secado, visualización e interpretación se realiza en aproximadamente dos horas. Por ello, la estabilidad considerada en este estudio corresponde a la estabilidad operativa durante el procedimiento analítico, no a almacenamiento prolongado de soluciones preparadas.

### Consideraciones éticas

El estudio no involucró seres humanos, animales de experimentación ni información clínica individual. Las muestras analizadas correspondieron a medicamentos comerciales empleados exclusivamente para el desarrollo y validación analítica de métodos de tamizaje semicuantitativo. Por la naturaleza del estudio, no se requirió consentimiento informado. El proyecto fue aprobado por el Comité Institucional de Ética en Investigación del Instituto Nacional de Salud (código EI-037-15).

## RESULTADOS

Se desarrollaron y validaron ocho métodos por CCF para el tamizaje semicuantitativo de IFAs en medicamentos sólidos orales. Las condiciones cromatográficas óptimas se presentan en la tabla 1, incluyendo concentración del IFA, fase móvil, sistema de detección, valor de Rf y resolución entre IFAs en las formulaciones multicomponente. Los valores de Rf se ubicaron dentro del intervalo preestablecido de 0,20 a 0,85 y permitieron identificar los IFAs evaluados. En las combinaciones multicomponente, la resolución cromatográfica fue igual o superior a 1,0 bajo las condiciones validadas.

**Tabla 1:** Condiciones cromatográficas óptimas de los métodos por cromatografía en capa fina (CCF) para los ingredientes farmacéuticos activos (IFAs) evaluados.

IFAs evaluados	Concentración del IFA	Fase móvil	Método de detección	Valor de Rf	Resolución entre IFAs
Aciclovir	200, 400 y 800 mg	Acetato de etilo/metanol/solución de amoniaco 25% (9/11/3 mL)	UV 254 nm	0,56	-
Dicloxacilina	250 y 500 mg	Acetato de etilo/agua/ácido acético glacial (15/2/3 mL)	UV 254 nm; vapores de yodo	0,70	-
Dimenhidrinato	50 mg	Tolueno/acetato de etilo/ácido acético glacial/metanol (8/10/1,5/1 mL)	UV 254 nm	0,60	-
Ibuprofeno	200, 400, 600 y 800 mg	Acetato de etilo/metanol/solución de amoniaco 25% (18/4/2 mL)	UV 254 nm	0,28	-
Diclofenaco sódico	25, 50 y 100 mg	Tolueno/acetato de etilo/ácido acético glacial (9/10/1 mL)	UV 254 nm; vapores de yodo	0,61	-
Diclofenaco sódico/paracetamol	50/500 mg	Tolueno/acetato de etilo/ácido acético glacial (9/10/1 mL)	UV 254 nm; vapores de yodo	DCF: 0,62; PCM: 0,33	3,5
Norfloxacino/fenazopiridina clorhidrato	400/100 y 400/50 mg	Acetato de etilo/metanol/agua/solución de amoniaco 25% (10/5/0,6/4,4 mL)	UV 254 nm; vapores de yodo	FZP: 0,85; NFX: 0,35	8,3
Clorfenamina maleato/fenilefrina clorhidrato/paracetamol	2/5/500 y 4/10/500 mg	Acetato de etilo/tolueno/metanol/solución de amoniaco 25% (14/2/3/1 mL)	UV 254 nm; UV 366 nm; vapores de yodo	CFM: 0,68; PCM: 0,53; FEC: 0,44	1,05; 2,16^a^

DCF: diclofenaco sódico; PCM: paracetamol; NFX: norfloxacino; FZP: fenazopiridina clorhidrato; FEC: fenilefrina clorhidrato; CFM: clorfenamina maleato. En dimenhidrinato, el método se orientó al marcador principal difenhidramina.

a En la combinación clorfenamina maleato/fenilefrina clorhidrato/paracetamol, los valores de resolución corresponden al orden FEC-PCM y PCM-CFM, respectivamente.

Las matrices evaluadas correspondieron a formulaciones comerciales disponibles en el mercado nacional. Se incluyeron cuatro matrices de aciclovir, siete de dicloxacilina, cinco de dimenhidrinato, diez de ibuprofeno, seis de diclofenaco sódico, diez de diclofenaco sódico/paracetamol, ocho de norfloxacino/fenazopiridina clorhidrato y siete de clorfenamina maleato/fenilefrina clorhidrato/paracetamol.

Durante la optimización se seleccionaron diluyentes que mantuvieron el comportamiento cromatográfico de las muestras durante la ejecución del ensayo. Considerando que la aplicación completa de las metodologías rápidas toma aproximadamente dos horas, las soluciones preparadas mostraron estabilidad operativa suficiente para el tamizaje dentro de esa ventana de trabajo. No se observaron cambios visibles que impidieran la lectura del Rf, la comparación de intensidad de manchas o la interpretación semicuantitativa durante el procedimiento analítico.

La preparación de soluciones madre se ajustó a la concentración del IFA declarado en cada medicamento, con el propósito de obtener soluciones intermedias comparables entre presentaciones comerciales. A partir de estas soluciones madre se prepararon soluciones de trabajo al 80%, 100% y 120% empleadas como referencias semicuantitativas para la lectura visual y densitométrica de las manchas.

En algunos métodos, el diluyente de la solución de trabajo fue distinto al utilizado en la solución madre, como ocurrió con aciclovir, cuya solución madre se preparó en NaOH 0,1 M y sus soluciones de trabajo se prepararon con metanol. Las condiciones de preparación se resumen en la tabla 2, mientras que las diluciones específicas para cada nivel de trabajo se presentan en la tabla suplementaria 1.

**Tabla 2:** Preparación de soluciones madre para muestras y materiales de referencia en los métodos por cromatografía en capa fina (CCF).

IFAs evaluados	Concentración del medicamento	Preparación de la solución madre	Concentración final de la solución madre	Diluyente
Aciclovir	200 mg	Triturar 1 tableta y disolver con 40 mL de diluyente	5 mg/mL	NaOH 0,1 M en agua
	400 mg	Triturar 1 tableta y disolver con 80 mL de diluyente	5 mg/mL	NaOH 0,1 M en agua
	800 mg	Triturar 1 tableta y disolver con 160 mL de diluyente	5 mg/mL	NaOH 0,1 M en agua
Dicloxacilina, como sal sódica	250 mg	Abrir 1 cápsula y disolver con 12,5 mL de diluyente	20 mg/mL	Metanol
	500 mg	Abrir 1 cápsula y disolver con 25 mL de diluyente	20 mg/mL	Metanol
Dimenhidrinato	50 mg	Triturar 1 tableta o abrir 1 cápsula y disolver con 10 mL de diluyente	5 mg/mL	Metanol acuoso al 90%
Ibuprofeno	200 mg	Triturar 1 tableta o abrir 1 cápsula y disolver con 20 mL de diluyente	10 mg/mL	Metanol
	400 mg	Triturar 1 tableta o abrir 1 cápsula y disolver con 40 mL de diluyente	10 mg/mL	Metanol
	600 mg	Triturar 1 tableta o abrir 1 cápsula y disolver con 60 mL de diluyente	10 mg/mL	Metanol
	800 mg	Triturar 1 tableta o abrir 1 cápsula y disolver con 80 mL de diluyente	10 mg/mL	Metanol
Diclofenaco sódico	25 mg	Triturar 1 tableta o abrir 1 cápsula y disolver con 12,5 mL de diluyente	2 mg/mL	Metanol
	50 mg	Triturar 1 tableta o abrir 1 cápsula y disolver con 25 mL de diluyente	2 mg/mL	Metanol
	100 mg	Triturar 1 tableta o abrir 1 cápsula y disolver con 50 mL de diluyente	2 mg/mL	Metanol
Diclofenaco sódico/paracetamol	50/500 mg	Triturar 1 tableta y disolver con 25 mL de diluyente	DCF: 2 mg/mL; PCM: 20 mg/mL	Metanol
Norfloxacino/fenazopiridina clorhidrato	400/100 mg	Triturar 1 tableta o abrir 1 cápsula y disolver con 80 mL de diluyente	NFX: 5 mg/mL; FZP: 1,25 mg/mL	Agua acidificada
	400/50 mg	Triturar 1 tableta o abrir 1 cápsula y disolver con 80 mL de diluyente	NFX: 5 mg/mL; FZP: 0,625 mg/mL	Agua acidificada
Clorfenamina maleato/fenilefrina clorhidrato/paracetamol	2/5/500 mg	Triturar 2 tabletas, abrir 2 cápsulas o disolver 2 sobres con 10 mL de diluyente	CFM: 0,4 mg/mL; FEC: 1 mg/mL; PCM: 100 mg/mL	Metanol
	4/10/500 mg	Triturar 2 tabletas, abrir 2 cápsulas o disolver 2 sobres con 10 mL de diluyente	CFM: 0,8 mg/mL; FEC: 2 mg/mL; PCM: 100 mg/mL	Metanol

DCF: diclofenaco sódico; PCM: paracetamol; NFX: norfloxacino; FZP: fenazopiridina clorhidrato; CFM: clorfenamina maleato; FEC: fenilefrina clorhidrato.

Agua acidificada: mezcla de 2 mL de ácido acético glacial con 78 mL de agua.

En el caso de dimenhidrinato, el método se orientó al marcador principal difenhidramina.

La solución madre fue empleada como solución intermedia para preparar las soluciones de trabajo al 80%, 100% y 120%. El diluyente indicado en esta tabla corresponde a la preparación de la solución madre; los diluyentes y volúmenes usados para las soluciones de trabajo se detallan en la Tabla suplementaria 1. En aciclovir, la solución madre se preparó en NaOH 0,1 M, mientras que las soluciones de trabajo se prepararon con metanol. En ibuprofeno, la solución madre de 10 mg/mL se empleó como intermediario para preparar la solución de trabajo al 100% de 8 mg/mL, definida como referencia operativa para la comparación semicuantitativa.

En la figura suplementaria 1 con fines demostrativos del desempeño cromatográfico del método, se muestra una placa de CCF de la combinación fenilefrina clorhidrato/paracetamol/clorfenamina maleato, empleada para ilustrar la separación cromatográfica de los tres IFAs bajo luz UV de 254 nm y 366 nm. En esta placa, los carriles rotulados como 80% y 100% corresponden a referencias de comparación semicuantitativa, y los carriles de muestra permiten observar la presencia o ausencia de las manchas correspondientes a los ingredientes evaluados.

La validación analítica se resume en la tabla 3. Todos los métodos cumplieron los criterios preestablecidos de linealidad, con coeficientes de correlación superiores a 0,99 para el sistema y el método. La especificidad/selectividad fue adecuada, ya que la diferencia relativa entre el Rf de la muestra y el Rf del estándar se mantuvo dentro del criterio de ≤5%, sin interferencias relevantes del diluyente, excipientes o componentes de matriz; en las formulaciones multicomponente, la resolución cromatográfica fue ≥1,0 bajo las condiciones validadas. La precisión, evaluada bajo condiciones de repetibilidad e intermedia, presentó coeficientes de variación dentro de los límites de aceptación; la exactitud, expresada como sesgo frente a HPLC, se mantuvo dentro del intervalo de ±3,00%, y la robustez demostró que las variaciones deliberadas de los factores evaluados no produjeron cambios significativos en los valores de Rf. En dimenhidrinato la interpretación se orientó a difenhidramina como marcador principal.

**Tabla 3:** Parámetros de validación analítica de los métodos por cromatografía en capa fina (CCF).

IFA evaluados	Especificidad/selectividad	Linealidad sistema r	Linealidad método r	Límite de detección visual (mg/mancha) ^a^	Repetibilidad CV%	Precisión intermedia CV%	Sesgo, IC 90%	Robustez
ACV	Cumple; Rf muestra/estándar ≤5%	0,9958	0,9966	0,050	1,53	4,89	-0,57 a 0,37	0,0465
DCX	Cumple; degradación próxima bajo hidrólisis ácida	0,9917	0,9966	6,400	1,68	3,63	0,01 a 0,51	0,0547
DMH	Cumple para difenhidramina como marcador	0,9966	0,9979	0,800	1,38	1,51	-1,15 a 0,37	0,0156
IBP	Cumple; Rf muestra/estándar ≤5%	0,9954	0,9995	3,200	1,77	4,86	-1,09 a 0,58	0,0199
DCF	Cumple; Rf muestra/estándar ≤5%	0,9942	0,9943	0,150	1,62	1,52	-0,25 a 0,83	0,0465
DCF en DCF/PCM	Cumple; resolución ≥1,0	0,9960	0,9984	0,150	1,20	4,45	0,00 a 1,19	0,0191
PCM en DCF/PCM	Cumple; resolución ≥1,0	0,9947	0,9970	0,750	1,86	3,77	-0,52 a 0,45	0,0414
FZP en FZP/NFX	Cumple; resolución ≥1,0	0,9972	0,9960	0,250	1,74	3,21	0,38 a 0,78	0,0190
NFX en FZP/NFX	Cumple; resolución ≥1,0	0,9968	0,9977	0,125	1,87	4,92	-0,17 a 0,61	0,0263
CFM en CFM/FEC/PCM	Cumple; resolución ≥1,0	0,9950	0,9965	0,120	2,33	2,40	-0,83 a 0,08	0,0517
FEC en CFM/FEC/PCM	Cumple; resolución ≥1,0	0,9960	0,9939	0,030	2,89	3,11	-0,30 a 0,58	0,0290
PCM en CFM/FEC/PCM	Cumple; resolución ≥1,0	0,9968	0,9945	0,018	2,72	2,76	-0,02 a 0,94	0,0198

r: coeficiente de correlación; CV: coeficiente de variación; IC: intervalo de confianza; ACV: aciclovir; DCX: dicloxacilina; DMH: dimenhidrinato; IBP: ibuprofeno; DCF: diclofenaco sódico; PCM: paracetamol; FZP: fenazopiridina clorhidrato; NFX: norfloxacino; CFM: clorfenamina maleato; FEC: fenilefrina clorhidrato.

a El parámetro reportado corresponde a límite de detección visual, no a límite de cuantificación formal.

El kit portátil Hampi Tariki se presenta en la figura 1. El diseño integra los materiales necesarios para ejecutar los métodos en una jornada corta de tamizaje: placas cromatográficas, cámaras de desarrollo, capilares, materiales de referencia, manual operativo, frascos de vidrio para el transporte de disolventes y dos lámparas portátiles de luz UV de 254 nm y 366 nm. Esta configuración permite realizar la lectura de las placas de acuerdo con el sistema de detección definido para cada IFA.

**Figura 1:** Diseño interior del kit portátil Hampi Tariki.

## DISCUSIÓN

En este estudio se desarrollaron y validaron ocho métodos por CCF para el tamizaje semicuantitativo de IFAs en tabletas y cápsulas comercializadas en el Perú. Los métodos permitieron diferenciar niveles equivalentes al 80%, 100% y 120% del contenido declarado mediante comparación visual y densitométrica del Rf, tamaño e intensidad de las manchas. Esta aproximación es coherente con el uso de la CCF como herramienta de tamizaje, más que como método confirmatorio de cuantificación ^8^^-^^13^.

Los resultados de especificidad/selectividad respaldan el uso de los métodos como pruebas preliminares de tamizaje, ya que los IFAs pudieron identificarse en matrices comerciales sin interferencias relevantes y con diferencias de Rf dentro del criterio de aceptación. Este punto es importante porque se evaluaron productos de marca y genéricos de distintos fabricantes, con posibles variaciones en excipientes. En los medicamentos multicomponente, la separación cromatográfica permitió diferenciar los IFAs bajo las condiciones validadas; sin embargo, estos resultados no deben extrapolarse a combinaciones no evaluadas. En dimenhidrinato, la interpretación se delimitó a difenhidramina como marcador principal, debido a que la 8-cloroteofilina presentó un Rf bajo y no fue validada como marcador independiente.

La linealidad fue satisfactoria para todos los IFAs evaluados, con coeficientes de correlación superiores a 0,99, lo que evidencia proporcionalidad entre la concentración y el área densitométrica de la mancha en el intervalo de trabajo. Este resultado respalda la utilidad del método para diferenciar niveles semicuantitativos, especialmente cuando el objetivo es identificar desviaciones marcadas respecto al contenido declarado.

La precisión fue adecuada bajo condiciones de repetibilidad y precisión intermedia, con coeficientes de variación dentro de los límites predefinidos. La participación de tres analistas en la precisión intermedia fortalece la aplicabilidad del método, dado que una prueba de tamizaje debe mantener un desempeño aceptable cuando es ejecutada por diferentes operadores. Asimismo, la evaluación de la robustez, mostró que las variaciones deliberadas evaluadas no produjeron cambios significativos en los valores de Rf, lo que sugiere estabilidad del comportamiento cromatográfico bajo pequeñas variaciones del procedimiento.

La exactitud, expresada como sesgo frente a HPLC, se mantuvo dentro del intervalo de aceptación definido. Esta comparación debe entenderse como una evaluación preliminar de concordancia analítica, dado que los métodos corresponden a pruebas semicuantitativas. En este sentido, su aplicación resulta pertinente como herramienta complementaria para la verificación rápida de la calidad de medicamentos, por lo que los métodos no deben presentarse como sustitutos de técnicas instrumentales confirmatorias ^10^^-^^13^.

La utilidad de la CCF para el tamizaje de medicamentos ha sido documentada previamente. Roy et al. Demostraron que los métodos simples por CCF pueden emplearse como pruebas iniciales de tamizaje, aunque no reemplazan los métodos oficiales de farmacopea ^8^. De forma similar, estudios con GPHF-Minilab han mostrado que esta tecnología puede identificar productos con ausencia del IFA, IFA incorrecto o contenido marcadamente bajo, especialmente en entornos con recursos limitados ^9^^,^^10^^,^^13^^,^^14^. Sin embargo, la literatura también señala que estos métodos tienen menor capacidad para detectar desviaciones moderadas del contenido o problemas de disolución, por lo que las muestras sospechosas requieren confirmación mediante métodos farmacopeicos o instrumentales ^10^^-^^15^.

La selección de los IFAs evaluados se relaciona con antecedentes nacionales de falsificación de medicamentos en formas sólidas orales y en grupos terapéuticos de uso frecuente, incluidos analgésicos, antiinflamatorios, antiinfecciosos y combinaciones sintomáticas ^1^^,^^2^. En ese contexto, los métodos desarrollados cubren un conjunto de productos pertinente para vigilancia sanitaria preliminar en el país, aunque su aplicación debe limitarse a las matrices y combinaciones evaluadas.

Una observación relevante para el uso operativo del kit es la estabilidad de las soluciones durante el ensayo. En este estudio, la selección del diluyente consideró no solo la solubilidad y recuperación del analito, sino también el comportamiento cromatográfico durante la ejecución del procedimiento. Dado que la aplicación completa de las metodologías rápidas toma aproximadamente dos horas, los resultados respaldan la estabilidad operativa de las soluciones durante esa ventana analítica. Esta evidencia no debe interpretarse como un estudio formal de estabilidad prolongada ni permite establecer tiempos de almacenamiento extendidos para soluciones preparadas.

El uso de frascos de vidrio para el transporte de disolventes constituye una característica operativa importante del kit. Esta elección reduce el riesgo de incompatibilidad entre los disolventes y los materiales de envase, una preocupación válida cuando se emplean tolueno o acetato de etilo. No obstante, para una implementación rutinaria en campo, será necesario documentar controles simples antes del uso, como verificación de cierre, ausencia de coloración anómala, ausencia de partículas y ausencia de evaporación visible. Estos controles no reemplazan un estudio formal de compatibilidad, pero permiten reducir riesgos operativos durante jornadas cortas de tamizaje.

La portabilidad del kit y la simplicidad relativa de la CCF pueden favorecer su uso como herramienta complementaria de vigilancia descentralizada. A diferencia de una descripción genérica de lectura instrumental, el kit Hampi Tariki incorpora dos lámparas UV independientes, una de 254 nm y otra de 366 nm, lo que permite realizar las lecturas de campo según el sistema de detección establecido para cada IFA. Además, el revelado con vapores de yodo representa una alternativa útil cuando la visualización UV no sea suficiente o cuando se requiera un sistema complementario de detección ^13^^,^^14^.

Entre las fortalezas del estudio se encuentran el desarrollo de métodos para varios IFAs de interés sanitario, la inclusión de formulaciones monocomponente y multicomponente, la evaluación de 57 matrices comerciales disponibles en el mercado nacional, el uso de criterios de aceptación previamente definidos y la comparación del sesgo frente a HPLC. Además, el estudio parte de una necesidad sanitaria documentada en el Perú, donde la falsificación afecta principalmente a medicamentos sólidos orales de alta circulación ^1^^,^^2^. En términos de salud pública, el kit Hampi Tariki podría contribuir a la vigilancia sanitaria descentralizada mediante la priorización de muestras sospechosas para su confirmación en laboratorios oficiales. Su valor potencial se encuentra en ofrecer una primera orientación analítica en escenarios donde se requiere decidir qué muestras deben enviarse con mayor prioridad a confirmación instrumental. En relación con el costo, el kit podría representar una alternativa operativamente accesible frente a tecnologías instrumentales o kit comerciales importados. Sin embargo, esta afirmación debe interpretarse como una ventaja potencial, dado que el presente estudio no incluyó una evaluación económica formal ni un análisis comparativo de costos frente a otras tecnologías de tamizaje ^(^^24^.

Este estudio también presenta limitaciones que deben reconocerse para contextualizar adecuadamente el alcance de los resultados. La validación se realizó con estándares de referencia y medicamentos comerciales previamente verificados como conformes, sin incluir muestras falsificadas reales, productos incautados ni formulaciones adulteradas. Por ello, los hallazgos no permiten estimar de manera directa el desempeño del kit frente a medicamentos falsificados presentes en el mercado. Otra limitación relevante es la ausencia de evaluación en condiciones de campo, donde factores como las variaciones ambientales, la iluminación disponible, la experiencia del operador y la logística de derivación de muestras pueden influir en el desempeño. Asimismo. La estabilidad documentada corresponde a la ventana operativa aproximada de dos horas durante la aplicación del método, sin considerar escenarios de almacenamiento prolongado de soluciones preparadas.

En conclusión, se desarrollaron y validaron ocho métodos por cromatografía en capa fina integrados en el kit Hampi Tariki para el tamizaje semicuantitativo de IFAS en medicamentos sólidos orales; bajo condiciones controladas de laboratorio, los métodos mostraron desempeño analítico adecuado y permitieron diferenciar niveles equivalentes al 80%, 100% y 120% del contenido declarado, por lo que constituyen una propuesta de tamizaje preliminar para identificar muestras sospechosas y priorizar su confirmación mediante HPLC u otros métodos oficiales, sin reemplazar a los procedimientos farmacopeicos confirmatorios.
